# Prognostic and Survival Factors in Myxofibrosarcomas

**DOI:** 10.1155/2012/830879

**Published:** 2012-06-11

**Authors:** Varun Dewan, Anna Darbyshire, Vaiyapuri Sumathi, Lee Jeys, Robert Grimer

**Affiliations:** ^1^Queen Elizabeth Hospital, Birmingham B15 2TH, UK; ^2^University of Southampton Medical School, Southampton SO16 6YD, UK; ^3^Royal Orthopaedic Hospital, Birmingham B31 2AP, UK

## Abstract

*Aim*. Our study aimed to determine prognostic factors for survival and recurrence in myxofibrosarcomas based on the experience of a single institution. *Methods*. Patients who had been diagnosed with a myxofibrosarcoma were identified from our database. Survival and recurrence were evaluated with Kaplan Meier survival curves for univariate and cox regression for multivariate analysis. *Results*. 174 patients with a diagnosis of myxofibrosarcoma were identified. Two patients were excluded due to incomplete information, leaving 172 patients with a mean age of 67 years. Surgery was undertaken in all but 6 patients. Five-year survival was better for myxofibrosarcomas when compared to other soft tissue sarcomas (63% versus 57%). Size, grade of tumour, age, and metastases were all found to be prognostic factors. Local recurrence occurred in 29 patients (17%) with an overall risk of 15% at 5 years. Previous inadvertent excision significantly raised this risk to 45%. Wide surgical margins and depth of tumour, however, had no impact on recurrence. *Conclusion*. Factors previously identified as prognostic did not demonstrate such a relationship in our study, highlighting the unpredictable nature of myxofibrosarcomas. Future treatment may lie in developing an understanding molecular basis of the tumour and directing therapies accordingly.

## 1. Introduction

The World Health Organisation describes myxofibrosarcomas as a spectrum of malignant fibroblastic lesions with variably myxoid stroma and pleomorphism, possessing a distinctively curvilinear vascular pattern [1]. They are one of the most common soft tissue tumours in the extremities of elderly patients and are notorious for their high rate of local recurrence. They are often higher grade than at presentation, therefore, leading to a greater risk of metastases [2], which is currently refractory to current nonsurgical treatment options [3]. Recurrence has been shown to occur in spite of repeated surgery involving wide local excision and negative surgical margins [4].

The aim of this study was to examine a large single institution experience to determine prognostic factors for survival and recurrence and compare this with the findings of other recently published large series from single institutions. In addition, comparison will also be made with the the results of myxofibrosarcomas and other types of STS at our institution.

## 2. Method

Since 1986, prospectively kept electronic patient records have been maintained at our institution, and as the diagnosis of myxofibrosarcoma was only popularised since the early 1990's, the first patient entered into this study was seen in 1993. Patients with a diagnosis of myxofibrosarcoma were identified and the size, grade, and depth of the tumour at presentation, recurrence, and mortality were reviewed for all of these patients.

A total of 174 patients were identified, of which complete information was available on 172 patients, which formed the study group. A total of 66 patients (38%) had undergone a previous inadvertent excision of a myxofibrosarcoma at another institution prior to referral to our unit, with the remaining 108 patients being newly diagnosed in our unit. The mean age at presentation was sixty-seven years old (range, fifteen to ninety-three years old). The mean maximum size of tumour excised was 8.4 centimetres (range, 0.3–31 cm). The majority of the tumours in the study population were histologically high grade (*n* = 87/172, 51%) and were deep to fascia in location (*n* = 93/172, 54%).

To assess the characteristics of myxofibrosarcomas, the dataset was analysed from a larger dataset of 2461 patients all histological subtypes of STS seen in our unit until 2007, which allowed comparison of demographic details, local recurrence and survival rates of patients with myxofibrosarcoma.

Survival and recurrence were evaluated with Kaplan Meier survival curves for univariate and cox regression for multivariate analysis. Factors assessed included size, site, location, margins, grade and depth were analysed.

## 3. Results

**Figure 1:**
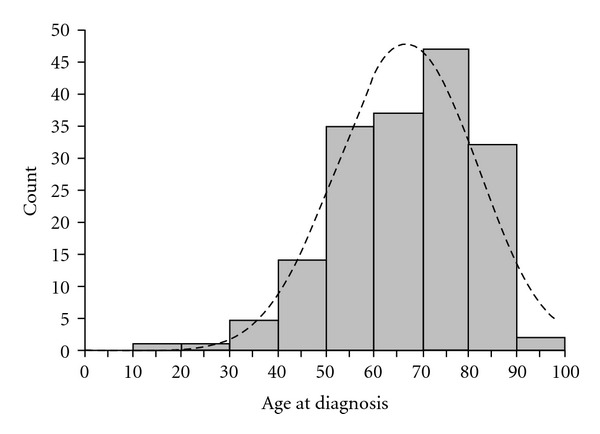
Age distribution histogram with normal distribution comparison showing a skewed population, being more frequent in the >75-year-old population.

The mean and median age of the study population was similar at 67 and 68 years respectively, with the mode being 78 years old, suggesting a skewed age distribution (Figure 1). The mean age was also significantly greater than the mean age for all subtypes of STS seen in our unit (67 years versus 53 years, *P* < 0.001). There was a significantly higher proportion of superficial tumours seen compared to all other histological subtypes of STS seen in our unit (46% versus 26%, *P* < 0.001). The proportion of tumours which were myxofibrosarcoma presenting with high grade tumours was significantly lower than other types of STS (51% versus 57%, *P* = 0.001) (Table 3). The group of myxofibrosarcoma patients were more likely to be distributed in the arm or leg than other STS subtypes.

**Figure 2:**
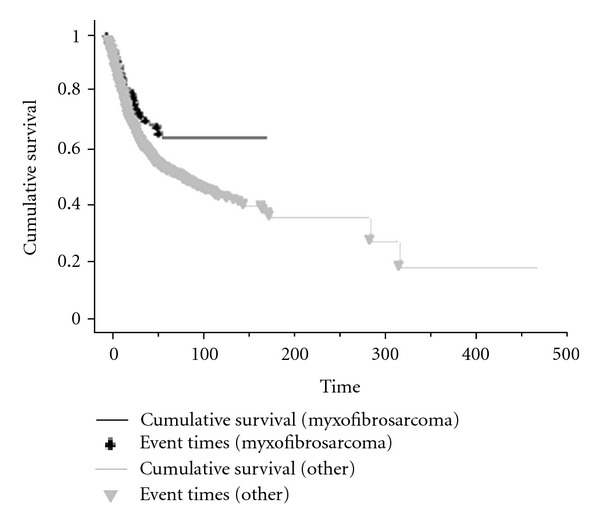
Kaplan Meier survival curve for myxofibrosarcoma compared with other STS with time in months from diagnosis (*P* = 0.01).

The overall survival of those patients with myxofibrosarcoma was significantly better than other types of STS, with 5-year survival being 63% versus 57% for all presentations (*P* = 0.01, Figure 2). Metastases were present at diagnosis in 6 patients (*n* = 6/174, 4%) and developed subsequently in 35 patients (*n* = 35/174, 20%). Metastases developed subsequent to locally recurrent disease in 12 patients (*n* = 12/35, 34%) and as an isolated event in 23 patients (*n* = 23/35, 64%) (Figure 4).

Locally recurrent disease occurred in 29 patients (*n* = 29/172, 17%) but was significantly more likely to occur if there was a previous unplanned excision of the tumour (cumulative risk of LR was 15% at 5 years compared to 45% following previous excision, *P* = 0.0009). Other univariate factors for development of locally recurrent disease were grade of tumour, where lower grade tumours were more likely to recur than high-grade tumours (LR rate for low grade = 22%, intermediate grade = 28%, high grade = 8%, *P* = 0.007) and anatomical site (*P* = 0.03) but interestingly wide surgical margins and depth of tumour were not prognostic for locally recurrent disease, even for those patients who were diagnosed and treated only in our unit. Patients who had undergone a previous unplanned excision had significantly lower grade tumours (*P* = 0.006), which were superficial (*P* = 0.008) and smaller (newly diagnosed had a mean size of 9.9 cm compared to 5.9 cm in the previously excised group, *P* < 0.001). On Cox regression multivariate analysis for development of locally recurrent disease the only prognostic factor was previous surgery (*P* = 0.03, HR = 5), with foot and ankle location tending to significance (*P* = 0.1, HR = 4).

**Figure 3:**
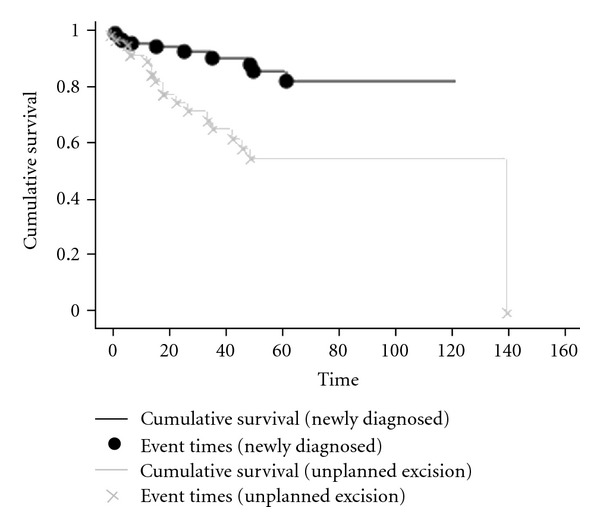
The effect of unplanned excision on locally recurrent disease, with the cumulative risk of LR rising from 15% at 5 years for those tumours primarily treated at our unit to 45% at 5 years for those treated with a previously unplanned sarcoma excision (*P* = 0.0009).

**Figure 4:**
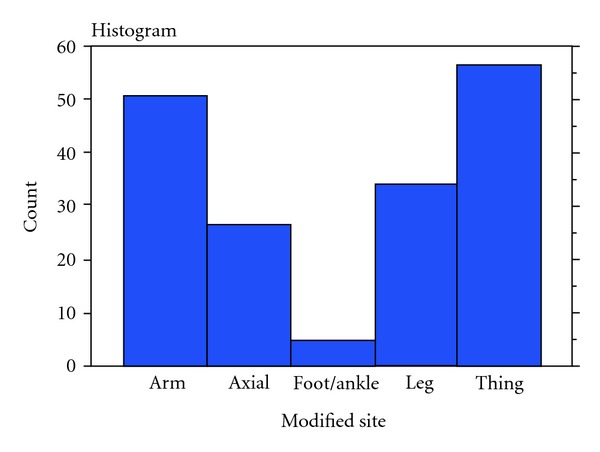
Histogram demonstrating location of myxofibrosarcomas.

The type of treatment was excision alone in 133 patients (73%), excision with split skin grafting in 26 patients (15%), excision and endoprosthetic replacement in one patient, and amputation in 6 patients (4%), and no definitive surgery was undertaken in 6 patients (4%) (Figure 3). Amputation was not more likely if the patient had undergone a previous excision. Surgical margins in the patients treated primarily in our unit were intralesional in 26%, marginal in 50%, and wide in 24%. Of the 21 patients with intralesional margins, 8 patients underwent further localised excision, achieving wide margins and 1 patient underwent an amputation, achieving a marginal margin, all those with intralesional underwent postoperative prophylactic radiotherapy. Local recurrence occurred in 5 patients with intralesional margins (24%), all of whom had undergone a further excision to extend the margins (Table 1).

The standard treatment was for postoperative radiotherapy (55 Gy) in all patients with deep, high-grade tumours, greater than 5 cm in size or those with poor margins. This was undertaken 6 weeks after operation and for all patients to have a scar reexcision and postoperative radiotherapy for those patients with a previous attempted excision before presentation to our unit (Table 2).

The only univariate prognostic factors for survival were grade of tumour (*P* = 0.01) and development of metastases (*P* > 0.0001). Locally recurrent disease, site, surgical margins, previous excision, and depth had no effect on survival. Independent prognostic factors on multivariate analysis for survival were not developing metastases (*P* = 0.002, HR = 0.39), age at diagnosis (*P* = 0.002, HR = 1.03), and size at resection (*P* = 0.01, HR = 1.1) (Table 1).

## 4. Discussion

Tumours now recognised as myxofibrosarcomas were initially described as the myxoid variant of malignant fibrous histiocytoma. It was not until the work of Angervall et al. [5] that the term myxofibrosarcoma was first used to describe this soft tissue tumour. Traditionally regarded as one of the most frequently encountered sarcomas in elderly patients, it is recognised by the World Health Organisation [1]. It is most commonly reported to occur in the extremities, whilst occasionally occurring in the trunk or head region, which is all supported by our data.

Myxofibrosarcomas are classified into two categories [3, 6]: superficial and deep. Superficial lesions are those regarded as being in the dermal or subcutaneous layer, whereas deep lesions are either intramuscular or subfascial. Superficial lesions tend to infiltrate, whereas deep lesions form a single discrete mass with a nodular appearance that spreads in a longitudinal manner [2]. Numerous studies have shown myxofibrosarcomas to have a higher rate of superficial lesions compared to other soft tissue sarcomas [4, 6, 7], which is supported by our data.

Local recurrence of myxofibrosarcomas is a widely reported phenomenon and has been reported as high as 61% [7], which is higher than that of other soft tissue sarcomas [8]. Our series, however, demonstrated that local recurrence rates for newly diagnosed tumours are similar to many soft tissue sarcoma subtypes. Often these lesions may be mistaken for other inflammatory processes such as myxoid liposarcomas, low-grade fibromyxoid sarcomas, cellular myxomas, and nodular fasciitis [4, 9]. This is demonstrated in our series, with a high rate of patients presenting who had had a previous inadvertent excision. These tumours were smaller, more superficial, and lower grade when compared to newly diagnosed patients. The difficulties in identifying these lesions, inevitably, impacted the ability to establish an initial accurate diagnosis. Current consensus is that the lack of pseudocapsule in the superficial subtype allows myxofibrosarcomas to exhibit the ability to extend along fascial planes to involve surrounding tissues resulting in inadequate excision. This goes someway to explaining the high rate of locally recurrent disease in the previous excision subgroup; as this group is more likely to have higher intralesional excision rate and a greater proportion of more superficial tumours. The deeper discrete tumours are, the easier they are to identify and less likely to be operated upon by a nonsarcoma specialist.

Various factors have been identified as being important in influencing recurrence rates: size, location, myxoid component, and age. Our data showed that the only statistically significant factor for local recurrence was when there had been a previous inadvertent excision. Our results show that those patients treated elsewhere or in our unit with an intralesional margin have high rates of local recurrence even when a wide margin is finally achieved with further re-excision. Nevertheless, our feeling remains that tumour-free surgical margins at first excision are important as have been previously demonstrated [9–11], but greater numbers are needed in our study to demonstrate significance.

As is typical for most soft tissue sarcomas: larger, high grade, and deep tumours have a poorer prognosis. Whereas, previous studies have shown that tumor grade is not associated with frequency of local recurrence [3, 4], our study shows that this is a univariate prognostic factor. Its ability to recur at higher grades is likely to be the cause of this. It has been postulated that myxofibrosarcomas gradually become more cellular, more pleomorphic, more mitotically active, and more necrotic [3]. The tendency of low-grade lesions to recur at higher grades underlines the need for accurate diagnosis and excision in specialist centres due to the potential for development of metastatic disease [3]. It should, however, be noted that our data, unlike other studies, showed that in the majority of cases, metastatic disease was a solitary disease relapse and was only subsequent to local relapse in approximately one-third of cases. Metastatic myxofibrosarcomas are frequently refractory to current treatment strategies and constitute the primary cause of sarcoma related death [3, 12].

The natural history of myxofibrosarcomas and its tendency to recur as higher grade lesions with a potential to metastise means that overall survival is likely to be linked to recurrence. The five- and ten-year survival rates demonstrated in our series (63% and 42% resp.) were comparable to that of other studies [6, 13]. Locally recurrent disease, however, was not an independent prognostic factor for survival in our series. Nevertheless, decreasing the rate of local recurrence is of paramount importance. The results clearly show a poorer rate of local control in the group of inadvertently excised tumours. This may be achieved by improving diagnosis, expediting early referral to a sarcoma centres and ensuring wide tumour-free surgical margins in superficial tumours. This in part involves better surgical planning through detailed imaging prior to surgery [14]. However, in order to develop new treatment strategies and improve survival in the long term, a greater understanding of the molecular basis of metastatic spread may help to achieve this.

Current work has focused upon the overexpression of MET and ezrin. MET, a cell receptor, represents the ligand for hepatocyte growth factor (HGF), which is responsible for epithelial cell dissociation, invasion, and angiogenesis [15, 16]. MET overexpression has been identified in numerous human tumours, and its tumorigenic properties have recently been demonstrated in osteosarcomas, where it is able to drive the transformation of osteoblasts into malignant cells [17]. The MET oncogene has also been shown to be influential in myxofibrosarcomas. It is predictive of shorter overall survival and metastasis-free survival as well as tumour size and mitotic rate [18]. As a result, this represents an appealing future therapeutic target and research has been targeted to achieve this goal. Cassinelli et al. [19] utilised mice to replicate a lung-metastasizing human tumor model demonstrating inhibition of MET activation and signaling in lung cell carcinomas. Thereby, demonstrating that MET targeting is a viable future therapeutic option in the treatment of metastatic cancer.

The exact upstream mechanism of action of MET in myxofibrosarcomas remains unclear, but it may be related to ezrin. Their relationship has previously been demonstrated. Overexpression of ezrin, a protein known to play a key role in cancer metastasis, has already been shown to result in disease progression and metastasis in a number of cancers including osteosarcomas [18] and rhabdomyosarcomas [20]. With respect to myxofibrosarcomas, Huang et al. demonstrated that ezrin overexpression was associated with higher tumor grade as well as being a poor prognosticator of disease-specific survival and metastasis-free survival. Hence, those tumours found to overexpress that ezrin should be monitored closely postoperatively [21].

Myxofibrosarcomas present a challenging management conundrum due to their unpredictable clinical course. Currently, ensuring tumour-free surgical margins is of paramount importance. However, the future of management of this tumor lies in developing a greater understanding of the molecular basis of this tumor to develop and direct therapy accordingly.

## Figures and Tables

**Table 1:** Prognostic factors for local recurrence and survival.

Local recurrence	
Univariate prognostic factors	Multivariate Prognostic Factors

Previous unplanned excision Lower grade tumours	Previous surgery

Survival	
Univariate prognostic factors	Multivariate Prognostic Factors

Grade of tumours Development of metastases	Not developing metastases Age at diagnosis Size at resection

**Table 2:** Patient and disease characteristics.

Characteristic	Value
Total	174
*Newly diagnosed *	*108 (62%) *
*Previous excision*	*66 (38%)*

Mean age (yrs)	67 (range: 15–93)

Depth	
*Superficial *	*79 (46%) *
*Deep*	*23 (13%)*

Mean tumour size (cm)	8.4 (range: 0.3–31)
*New diagnosis *	*9.9 *
*Previous excision*	*5.9*

Grade	
*High *	*87 (51%) *
*Intermediate *	*62 (36%) *
*Low*	*23 (13*%)

Metastases	41 (24%)
*At diagnosis *	*6 (3%) *
*Following diagnosis*	*35 (20%)*

Local recurrence at 5-year cumulative risk	
*Planned excision *	15%
*Unplanned excision*	45%

Risk of local recurrence by tumour grade	
*Low *	*22% *
*Intermediate *	*28% *
*High*	*8%*

Surgical margins	
*Wide *	*24% *
*Marginal *	*50% *
*Intralesional*	*26%*

**Table 3:** Myxofibrosarcoma versus other soft tissue sarcomas.

Characteristic	Myxofibrosarcoma	Other Soft tissue sarcomas
Mean age (yrs)	67	53
Superficial tumours (%)	46	26
Presenting with a higher grade tumour (%)	51	57
5-year survival (%)	63	57
